# Directed Communication in Theta and Alpha Networks Supports Content Handling in Working Memory

**DOI:** 10.1002/hbm.70537

**Published:** 2026-04-29

**Authors:** Julia Elmers, Moritz Mückschel, Christian Beste

**Affiliations:** ^1^ Department of Child and Adolescent Psychiatry, Faculty of Medicine Cognitive Neurophysiology, TUD Dresden University of Technology Dresden Germany

**Keywords:** alpha, directed connectivity, EEG, mental rotation, theta, working memory

## Abstract

Executive functions enable flexible control of behavior by dynamically coordinating perception, memory, and action. Among them, working memory plays a central role by maintaining and transforming information to meet current goals. Here, we examined mental rotation—a core operation that exemplifies these control dynamics—and delineated the role of directed communication in theta and alpha band activity in cortical networks. Replicating the typical behavioral pattern in mental rotation, we showed that high‐demand rotations were characterized by decreased theta and increased alpha power, reflecting a functional reallocation from control‐intensive monitoring to stabilized maintenance of visual representations. EEG‐beamforming localization identified anterior temporal, inferior frontal, and insular regions showing stronger directed information transfer to temporo‐parietal and occipito‐temporal regions. These findings suggest that mental rotation is associated with frequency‐specific, hierarchically organized, directed communication between anterior control‐related and posterior representational systems. In this directed communication, theta band dynamics likely coordinate working memory updating and response selection, whereas alpha‐band coupling stabilizes mnemonic representations through inhibitory gating. The study suggests that directed theta and alpha dynamics may support the flexible transformation of internal representations in working memory.

## Graphical abstract

We examined mental rotation and delineated the role of directed communication in theta and alpha band activity in cortical networks.

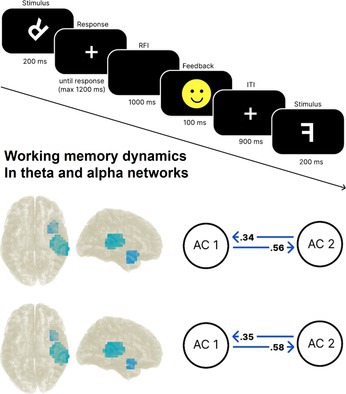

## 1 Introduction

Cognitive control relies on a set of executive functions that orchestrate perception, memory, and action in a goal‐directed manner (Diamond 2013). Within this framework, working memory constitutes a central mechanism that allows for the temporary storage and dynamic transformation of information. Mental rotation tasks provide a classic means to study these processes, as they require the maintenance and manipulation of visual representations in real time (Ebert et al. 2025). It comprises several interdependent aspects, including the visual recognition and encoding of an object, the manipulation and maintenance of its rotated representation in the working memory, and subsequent decision‐making processes such as determining whether objects are identical or mirrored, which is often followed by a motor response (Gogos et al. 2010; Prescott et al. 2010; Tomasino and Gremese 2015). The successful coordination of these subprocesses is likely supported by brain‐wide distributed neural systems, including the parietal cortex and premotor and supplementary motor areas (Cona, Marino, and Semenza 2017; Cona, Panozzo, and Semenza 2017; Cona and Scarpazza 2019; Mizuguchi et al. 2013; Veldema et al. 2021; Vingerhoets et al. 2001; Zacks 2008). Activity within the intraparietal sulcus (IPS), in particular, has been shown to increase proportionally with the angular disparity of to‐be‐rotated objects, highlighting its role in the spatial transformation of mental representations (Gogos et al. 2010; Zacks 2008). Beyond parietal and precentral contributions, mental rotation also engages midfrontal and temporal regions (Cona and Scarpazza 2019; Hawes et al. 2019; Tomasino and Gremese 2015; Veldema et al. 2021; Vingerhoets et al. 2001; Zacks 2008). Such ventral visual stream areas probably support processes of perceptual representation and visual memory (Zimmer 2008).

On a neurophysiological level, especially alpha and theta oscillations support mental rotation. Thereby, a decrease in alpha band power (8–12 Hz) is a consistent marker of cortical activation in mental rotation (Nikolaev and Anokhin 1998; Pineda 2005) and also in memory encoding, retrieval, and integration (Hanslmayr et al. 2012, 2014; Klimesch 2012; Klimesch et al. 1997; Zhu et al. 2019). Recent research suggests that alpha may serve as a gating mechanism that facilitates processing in task‐relevant cortical areas by tracking content‐specific working memory capacity and suppressing task‐irrelevant features (Chen et al. 2022; Wang, van Driel, et al. 2019). This decrease in alpha power might reflect a more distributed neuronal recruitment and attentional engagement (Pineda 2005) to allow efficient processing (Hanslmayr et al. 2012, 2016). The importance of theta band activity (4–7 Hz) for mental rotation performance (Heil 2002; Krause et al. 2021) is likely due to its role in working memory retrieval (Beste et al. 2023; Hsieh and Ranganath 2014; Jensen and Tesche 2002; Rempel et al. 2021; Wendiggensen and Beste 2023). In addition, theta activity is associated with the synchronization of neural activity between distant cortical regions (Buzsáki 2006; Buzsáki and Draguhn 2004; Cavanagh and Frank 2014; Cohen 2014) involved in working memory. So far, results from functional EEG connectivity analyses showed that larger angular disparities of to be rotated objects (e.g., 180° compared to 60° rotations) are associated with increased synchronization between prefrontal, parietal, and occipital sites (Silberstein et al. 2003). However, it remains unclear how directed communication/information transfer between cortical regions is modulated in the theta and alpha frequency bands during the integration of various subprocesses of mental rotation. Therefore, key mechanistic elements from a cognitive science perspective that explain how mental rotation arises have not yet been linked to directed communication within distinct oscillatory networks. However, this link is essential for developing mechanistic explanations of how different oscillatory activities (e.g., in the alpha and theta bands) support mental rotation, and, ultimately, visuospatial working memory processes.

In the current study, we used a data‐driven artificial neural network approach, the so‐called nonlinear Causal Relationship Estimation by Artificial Neural Network (nCREANN) method (Talebi et al. 2019) to estimate linear and nonlinear directed connectivity measures between cortical theta and alpha band activity networks. Compared to traditional research, nCREANN can capture both, linear and nonlinear dynamics of a network, which is crucial given the fact that brain functions are inherently nonlinear (e.g., neural firing, synaptic plasticity, neurotransmitter modulation) and previous findings highlight the importance of nonlinear connections (Azarmi et al. 2023; Motlaghian et al. 2022; Stam 2005), especially in perception–action integration processes relevant to cognitive control (Jamous et al. 2024; Talebi et al. 2024; Wang et al. 2024) and working memory dynamics (Elmers et al. 2024). Given this framework, we aimed to investigate how directed oscillatory communication in theta and alpha networks supports the integration of perceptual, mnemonic, and motor processes during mental rotation. Previous neuroimaging and electrophysiological work suggest that anterior temporal, inferior frontal, and insular regions form a multimodal integration hub that transforms perceptual input into higher‐order action concepts, whereas temporo‐parietal and temporo‐occipital regions subserve stimulus encoding, visuospatial transformation, and response selection. We therefore hypothesized that mental rotation relies on frequency‐specific, directionally organized networks linking these regions.

In the theta band (4–7 Hz) we expected directed information flow from anterior temporal and inferior frontal cortices, including the insula, towards inferior parietal regions. This anterior‐to‐posterior coupling is likely to reflect top‐down modulation of monitoring and response selection processes under increased cognitive demands. In contrast, in the alpha band (8–12 Hz) we anticipated directed connectivity from fronto‐temporal areas to posterior temporo‐occipital cortices along, corresponding to the transmission of mnemonic and perceptual templates for object recognition and mental rotation. Finally, we explored whether nonlinear directed connectivity, as captured by the nCREANN approach, would more sensitively detect these top‐down control pathways than linear models.

Together, these analyses aimed to delineate whether anterior temporal–frontal–insular systems in concert with posterior cortical regions, and through theta‐ and alpha‐band dynamics, support visuospatial transformations within working memory.

## 2 Methods

### 2.1 Participants

Data from *N* = 55 healthy participants (35 female, mean age = 33.8 ± 9.8 years; all right‐handed) were included in the study. All participants reported normal or corrected‐to‐normal vision and hearing and no history of psychiatric or neurological disorders. Written informed consent was obtained from all individuals prior to participation. The study was approved by the Ethics Committee of TU Dresden (EK 219062018) and conducted in accordance with the Declaration of Helsinki. Four participants were excluded from EEG analyses blind to final results from the analyses due to insufficient data quality (i.e., fewer than 15 valid segments in the conditions of interest) and one due to a missing logfile, resulting in a final EEG sample of *N* = 50 (32 female, mean age = 33.5 ± 9.9 years). The sample size of *N* = 50 is line with previous studies applying similar methods for directed connectivity analyses (Elmers et al. 2024; Mayer et al. 2025; Talebi et al. 2024) and behavioral task performance (Yu et al. 2022). For the behavioral analyses, two participants were excluded due to missing logfiles and three due to extreme outliers in reaction times (RTs) and/or hit rates in the conditions of interest. However, since behavioral data was sufficient for two participants, whose EEG files were excluded, we kept these participants in the behavioral analysis on behalf of statistical power, yielding a final behavioral sample of *N* = 50 (32 female, mean age = 33.6 ± 9.7 years).

### 2.2 Task and Procedure

**FIGURE 1:**
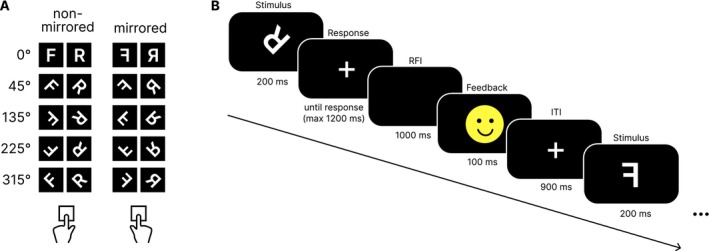
The Mental Rotation Task. (A) shows all stimuli displayed in the paradigm. (B) shows an example of one full trial with a non‐mirrored letter “R” rotated by 225° with positive feedback, and the stimulus of a following trial (letter “F”, mirrored, rotation angle 0).

Participants were seated in a dimly lit room, 60 cm away from a 24‐in. display screen with an eye‐tracking device. The Mental Rotation Task consisted of 20 stimuli (see Figure 1A). Two letters (i.e., “F” or “R”) were rotated clockwise with the following angles: 0°, 45°, 135°, 225°, or 315°. Letters were shown non‐mirrored or mirrored. Participants should press the left key for non‐mirrored and the right key for mirrored stimuli, independent of letter and rotation angle. Figure 1B shows an excerpt of the task. Each trial began with the display of the target stimulus for 200 ms. Participants had a maximum time of 1400 ms for responding, with time of response, a response‐feedback interval (RFI) of 1000 ms started. Subsequently, a yellow smiley face occurred if participants were fast enough, while a red frowny face appeared as a consequence of too slow or no responses. This feedback lasted for 100 ms and was followed by a fixed inter‐trial‐interval (ITI) of 900 ms showing a fixation cross. Prior to the experiment, participants completed 80 learning trials to familiarize with the experiment. The main task consisted of 640 trials (i.e., 8 blocks á 80 trials each). The stimuli were presented in a randomized order, with the frequency of rotation angles and mirrored versus non‐mirrored letters balanced across each block. The procedure is similar to previous studies by our group (Bensmann et al. 2019; Chmielewski et al. 2017; Hoffmann et al. 2014; Yu et al. 2023).

### 2.3 EEG Recording and Preprocessing

EEG data were collected with a 60‐channel Ag/AgCl EasyCap, featuring an equidistant electrode arrangement across the scalp. The reference electrode was positioned at Fpz, while the ground electrode was placed between AFz, Fz, and AF4 (without a fixed location). Signals were sampled at 500 Hz using either a QuickAmp or a BrainAmp DC/ExG amplifier (Brain Products GmbH, Gilching, Germany).

EEG signals were preprocessed using Automagic (Pedroni et al. 2019) and EEGLAB (Delorme and Makeig 2004) on Matlab 2020a (The MathWorks Corp.). In preparation for the Automagic pipeline, EEG datasets from BrainVision Analyzer were imported to EEGLAB format. Subsequently, raw EEG signals were downsampled to 256 Hz. Following this, flat channels (i.e., activity below 5 μV for more than 10,000 ms) were removed. In the last stage, the data were re‐referenced with the average reference method and removed channels were interpolated. The prepared data were then used for the main Automagic analysis. In the following, all settings made in the Automagic GUI are described: For the section “Bad Channel Detection”, we selected the PREP (Bigdely‐Shamlo et al. 2015) and the EEGLAB Clean_rawdata() pipeline to remove irregular artifacts and detect noisy channels. The PREP pipeline removes line noise, while the Clean_rawdata() pipeline applies a FIR high pass filter. For this, we used a transition band of 0.25 to 0.75, which yields a high pass filter of 0.5 Hz, line noise was set to 4, the channel criterion to 0.85. Segments showing abnormally strong power (> 15 standard deviations relative to calibration data) were reconstructed using Artifact Subspace Reconstruction (ASR; burst criterion: 15; Mullen et al. 2013). Time windows that could not be reconstructed were removed. Further, a residual bad channel detection was conducted with the following parameter settings: high variance 25, cutoff 100, rejection ration 0.5 and minimum variance 1. For the section “Artifact correction”, we selected EOG regression (EOG channels were FP1, AF7, FP2, AF8). Muscle and remaining eye artifacts were automatically classified and removed by using an independent component analysis (ICA) based Multiple Artifact Rejection Algorithm (MARA; (Winkler et al. 2011, 2014)). The box IClabel was not selected as it was used in a later step to remove remaining cardiac artifacts. We saved all components and selected the temporary high pass filter with a cutoff of 1 and a default filter order. In the “Filtering” section, we selected the line power frequency of 50 Hz (European standard), but did not select the Notch Filter as line noise is removed by PREP. Further, we selected pop_eegfiltnew() and set the low pass filter to 40. Other parameters were not selected in this section. Automagic creates quality ratings for every dataset. For the section “Quality Rating”, we chose the following parameters: Channel Threshold SD (mV) [10 20 30 40 50], Overall Threshold (mV) [20 25 30 35 40], and Time Threshold SD (mV) [10 20 30 40 50]. Lastly, in the section “Options”, only the box Detrending was selected and the down‐sampling rate was set to 2.

For the final step, several post‐processing steps of the Automagic output were conducted. First, since about 10% of datasets show cardiac artifacts after MARA, we applied IClabel (Pion‐Tonachini et al. 2019) with a threshold of 0.8 to select independent components (ICs) with cardiac artifacts and subsequently reject these. In the case of remaining cardiac artifact after IClabel, we did not further process these since these artifacts are usually not stimulus or response locked and therefore should cancel out when averaging trials; the energy of remaining artifacts usually is quite low, the affected channels (P11 and P12) are usually not used for analysis, and the spectral properties of artifacts should not affect theta or alpha or beta frequency band analysis significantly. Furthermore, a subsequent visual review of the data revealed that, if at all, remaining cardiac artifacts resulted in only minimal deviations. Second, all missing and “NaN” channels were interpolated using a spherical method since the Automagic GUI does not interpolate the EOG channels. Lastly, data segments removed by ASR in a previous step were readded to the dataset, removed samples were restored with value 0, and all event markers associated with these samples were restored. The restored data segments were marked with “Bad segment” in the event table list.

After Automagic preprocessing, data were imported to Brain Vision Analyzer 2.2 (Brain Products GmbH, Gilching, Germany) and segmented into target‐locked epochs. Each epoch comprised a 5‐s time window (i.e., 2000 ms pre‐target and 3000 ms post‐target onset). Afterwards, a baseline correction based on the activity from 200 ms pre‐target till target onset was applied. A flowchart of the analyzing pipeline can be found in the (Figure S1).

### 2.4 EEG Time‐Frequency Analysis and Source Localization

We employed an analysis pipeline that has previously been used in tasks investigating cognitive control (Elmers et al. 2024, 2025). For the EEG analyses, we pooled the data regarding the factor “mirroring” to reduce complexity in the analyses. Since behavioral results did not show significant differences between RTs and hit rates in trials between rotation angles 135° and 225°, we focused on differences between the most extreme rotation angles (i.e., 0° and 135°/225°).

Matlab 2020b (The MathWorks Corp.) and the FieldTrip toolbox (Oostenveld et al. 2011) were used for all EEG analysis. To assess power differences in theta and alpha band frequencies in the two rotation conditions (i.e., 0° and 135°), we first performed time‐frequency analyses of EEG data across a time frame of 3 s around stimulus onset [−1, 2]. After the time‐frequency analysis, we looked for significant differences between the conditions in the alpha and theta bands, respectively, using cluster‐based permutation tests (CBPT) with 500 permutations using the Monte Carlo approach. Thereby, time and frequency parameters were averages, hence clustering did only occur over electrodes. For clustering, the distance method was used, which draws a circle of certain size around each sensor‐position. Each other sensor included in this circle is defined to be a neighboring sensor. The alpha level for significance testing was set to *p* = 0.05 (2‐sided). The results of the earlier time frequency analyses (i.e., the examination of power differences in theta and alpha) were used to determine the CBPT time‐windows. Importantly, the results of the CBPTs only show whether there is a significant difference between the two conditions based on a broad cluster within all‐time points tested.

Subsequently, we applied the dynamic imaging of coherent sources (DICS) beamformer (Gross et al. 2001) together with a MNI‐based forward model (Holmes et al. 1998) to localize neural generators of oscillatory activity within the theta and alpha frequency bands. The DICS algorithm constructs a spatial filter from the sensor‐level cross‐spectral density (CSD) matrix, which allows frequency‐specific estimation of source power and coherence by suppressing contributions from non‐target sources. This approach provides direct estimates of functional interactions between brain regions in the frequency domain and has been demonstrated to yield high spatial accuracy for oscillatory source localization, making it particularly suitable for studies of neural synchrony (Halder et al. 2019; Mückschel et al. 2016). For the following connectivity analyses, a target‐locked time window from 0 to 1000 ms was selected for the DICS source localization for both frequency bands, while we used distinct time windows for theta and alpha frequency bands for the contrast analyses. Spectral estimates of power and the corresponding cross‐spectral density (CSD) matrices were obtained using a Hanning taper. After realigning the EEG electrode positions with the forward model, the brain volume was segmented into a 5 mm grid, and a leadfield matrix was computed for each grid point to enable spatial filtering. A common spatial filter with 5% regularization was applied across the two rotation conditions. Source estimates were normalized using a spatially inhomogeneous noise model based on the smallest eigenvalue of the CSD matrix to control for a central‐head noise bias (Van Veen et al. 1997). Following DICS beamforming, the top 2% of active source voxels were selected and grouped into clusters using the Density‐Based Spatial Clustering of Applications with Noise (DBSCAN) algorithm (Adelhöfer et al. 2020; Ester et al. 1996). For clustering, an epsilon value of 1.5 times the grid size (i.e., the length of each voxel) was used to include edge‐adjacent voxels. The minimum size of a cluster was set to at least three voxels. All voxels within each clusters were labeled according to the Automated Anatomical Labeling atlas (Rolls et al. 2020). This source localization pipeline was also applied for the contrast between rotation conditions. To avoid confusion, we will refer to contrast clusters (CCs), which were derived during contrast analyses and activity clusters (ACs) for analyses of directed connectivity.

### 2.5 Directed Connectivity Analysis

In the next step, we used the Linearly Constrained Minimum Variance (LCMV) beamformer (Van Veen et al. 1997) to reconstruct the virtual neural time series (from 0 to 1000 ms) for each voxel in each AC as a basis for the directed connectivity analysis with nCREANN. For this, a common spatial filter was derived with LCMV and multiplied with the EEG time‐domain data for each voxel. For each frequency band, a bandpass filter (windowed sinc FIR filter) was subsequently applied and voltage values were averaged across voxels separately for each AC in the separate conditions.

The analyses of directed connectivity were conducted separately for both frequency bands. For this, we used nCREANN (Talebi et al. 2019), a machine learning‐based approach which utilizes a non‐linear Multivariate Autoregressive (nMVAR) model together with an artificial neural network (ANN) to evaluate directed information transfer between ACs. MVARs assesses whether the prediction of a signal A based on its own past is enhanced by incorporating the past of another signal B. If the prediction is improved, it is assumed that B has a causal influence on A. Unlike conventional linear methods, which rely exclusively on linear MVAR models, nCREANN can account for both, linear and nonlinear dynamics, which has been shown to be important for working memory and cognitive control processes (Elmers et al. 2024; Wang et al. 2024; Yang et al. 2018). nCREANN was applied to the virtual neural time series derived from the LCMV beamforming step. To ensure sufficient data length for training of the ANN, single‐trial source signal values were concatenated. The optimal model order for each nCREANN analysis was selected based on the Akaike (AIC) and Schwartz criteria (SBC) using the ARfit toolbox (Schneider and Neumaier 2001). The model order thereby describes the time‐lag, which defines the number of past samples of the signal that serve as input values for the ANN. The smallest lag p at which the AIC and SBC values began to flatten (i.e., where further decreases in AIC/SBC became marginal, indicating adding more parameters no longer gave meaningful improvements in fit) was selected as the optimal model order, and this selection was applied consistently across subjects and conditions. The optimal model order (i.e., previous data points used to train the ANN) for virtual sensors in the theta frequency band was p = 7 and in the alpha frequency band p = 5 (see Figure S2). Since the ANN needs training data for fitting, no single subject evaluation was possible, hence optimal model order was derived on group level data and applied for all participants.

In a final step, the estimated directed connectivity values were normalized by dividing each subject's connectivity values by the maximum observed value across both conditions, separately for each linear and nonlinear connectivity measure (i.e., resulting in values ranging from 0 to 1). To test for significance of the connectivity values, 100 surrogate data sets with time‐shifted surrogate method were generated (Andrzejak et al. 2003; Papana et al. 2013). Therefore, all connections shown in the following reflect significant values of directed information transfer.

### 2.6 Statistical Analyses

Behavioral data analysis was conducted using IBM SPSS Statistics version 29.0.0.0. We calculated averaged reaction times (RTs) and hit rates for all 20 conditions (see Figure 1A) and each participant. Subsequently, we pooled RT and accuracy measures for letters, since we were mainly interested in the factors of rotation angle and mirroring. We ran a 2 × 5 repeated‐measures ANOVA with the within‐subject factors *Mirroring* (non‐mirrored vs. mirrored) and *Rotation* (0°, 45°, 135°, 225°, or 315°). A Greenhouse–Geisser correction was applied when assumption of sphericity was violated (i.e., tested with Mauchly test). Bonferroni‐correction was applied where necessary. To test, if directed connectivity values resulting from the nCREANN algorithm differed significantly between two ACs (e.g., asymmetric information transfer), we applied *t*‐tests with false discovery rate (FDR) correction to prevent an accumulation of Type I errors in multiple comparisons. Further, we used paired sample *t*‐tests to compare normalized averaged connectivity values conditions (e.g., nonlinear 0° vs. nonlinear 135° in theta network) and frequencies (e.g., nonlinear 0° in theta vs. alpha network).

## 3 Results

### 3.1 Behavioral Results

Regarding RTs, the repeated measures ANOVA revealed a main effect of Mirroring (F(1, 49) = 14.994, *p* < 0.001, partial *η* ^2^ = 0.234) with higher RTs in mirrored trials (509.49 ± 110.52 ms), compared to non‐mirrored trials (487.33 ± 88.65 ms). Furthermore, a main effect of Rotation was observed (F(1.544, 75.641) = 247.921, *p* < 0.001, partial *η* ^2^ = 0.835) and the interaction between factors Mirroring and Rotation was also significant (F(2.609, 127.833) = 3.501, *p* = 0.022, partial *η* ^2^ = 0.67). Since these effects violated the assumption of sphericity, a Greenhouse–Geisser correction was applied (*ε* for Rotation = 0.386; *ε* Mirroring×Rotation = 0.652).

**FIGURE 2:**
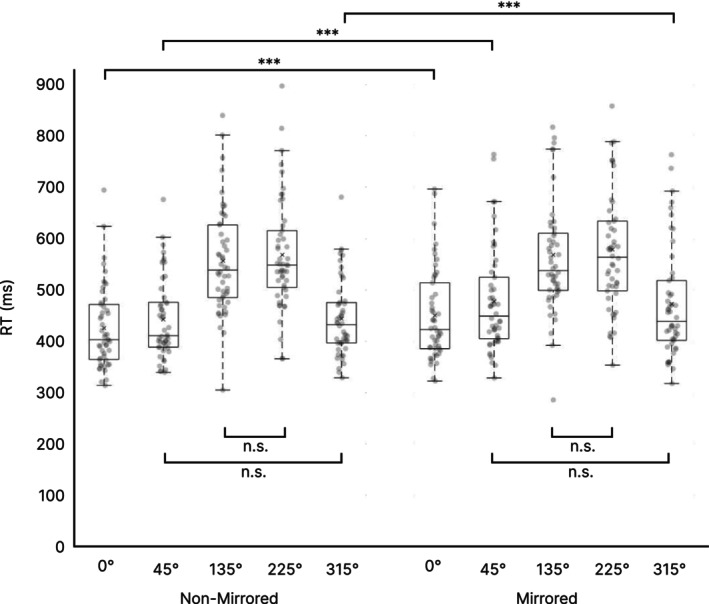
Behavioral RT results. Boxplots of RTs from the Mental Rotation Task are shown for non‐mirrored and mirrored trials for all rotation angles. For reasons of clarity, only significant differences between conditions and non‐significant differences within conditions are shown. ***p < 0.001, n.s. = non‐significant. Centerline = median; × = mean; box limits = upper and lower quartiles, whiskers = minimum and maximum within 1.5× interquartile range, dots = individual data points.

Post hoc paired‐sample *t*‐tests were conducted to evaluate differences between RTs in different rotation angles. To account for multiple comparisons, a Bonferroni correction was applied. In the non‐mirrored condition, no significant differences were found between equivalent rotation angles (i.e., 45°/315° and 135°/225°). All other comparisons were significant (see Figure 2 and Table 1). In the mirrored condition, we observed the same pattern (see Figure 2 and Table 1). Therefore, a gradual slowing with greater rotation angles till 135°/225° was observed in both mirroring conditions. We further compared the same rotation angles in the non‐mirrored and mirrored condition with paired‐sample *t*‐tests, and a Bonferroni correction was applied. Thereby, RTs differed significantly between low demanding rotation angles (0°: *t*(49) = −4.581, *p* < 0.001, *d* = −0.648; 45°: *t*(49) = −5.402, *p* < 0.001, *d* = −0.764; 315°: *t*(49) = −4.017, *p* < 0.001, *d* = −0.568), with faster RTs in the non‐mirrored condition compared to the mirrored condition. RTs in high demanding rotation angles (i.e., 135° and 225°) did not differ in non‐mirrored vs. mirrored trials (see Figure 2).

**TABLE 1:** Descriptive RT measures and paired‐sample t‐tests for non‐mirrored and mirrored trials (*N* = 50).

Non‐Mirrored		Mean (ms)	SD (ms)	*t*‐test
Pair 1	0°	425.84	80.01	*t*(49) = −6.696, *p* < 0.001, d = 0.947
	45°	442.46	78.24	
Pair 2	0°	425.84	80.01	*t*(49) = −15.641, *p* < 0.001, d = 2.212
	135°	556.16	103.08	
Pair 3	0°	425.84	80.01	*t*(49) = −17.196, *p* < 0.001, *d* = 2.432
	225°	567.93	107.35	
Pair 4	0°	425.84	80.01	*t*(49) = −5.709, *p* < 0.001, *d* = 0.807
	315°	444.27	74.56	
Pair 5	45°	442.46	78.24	*t*(49) = −13.833, *p* < 0.001, *d* = 1.956
	135°	556.16	103.08	
Pair 6	45°	442.46	78.24	*t*(49) = −16.190, *p* < 0.001, *d* = 2.290
	225°	567.93	107.35	
Pair 7	45°	442.46	78.24	*t*(49) = −0.589, *p* = 0.558
	315°	444.27	74.56	
Pair 8	135°	556.16	103.08	*t*(49) = −1.680, *p* = 0.099
	225°	567.93	107.35	
Pair 9	135°	556.16	103.08	*t*(49) = 15.141, *p* < 0.001, *d* = 2.141
	315°	444.27	74.56	
Pair 10	225°	567.93	107.35	*t*(49) = 17.761, *p* < 0.001, *d* = 2.512
	315°	444.27	74.56	
Mirrored		Mean (ms)	SD (ms)	*t*‐test
Pair 1	0°	450.51	88.55	*t*(49) = −8.521, *p* < 0.001, *d* = 1.205
	45°	477.51	101.05	
Pair 2	0°	450.51	88.55	*t*(49) = −13.112, *p* < 0.001, *d* = 1.854
	135°	568.69	126.59	
Pair 3	0°	450.51	88.55	*t*(49) = −12.803, *p* < 0.001, *d* = 1.811
	225°	578.54	128.74	
Pair 4	0°	450.51	88.55	*t*(49) = −4.974, *p* < 0.001, *d* = 0.703
	315°	472.21	107.65	
Pair 5	45°	477.51	101.05	*t*(49) = −12.301, *p* < 0.001, *d* = 1.740
	135°	568.69	126.59	
Pair 6	45°	477.51	101.05	*t*(49) = −11.617, *p* < 0.001, *d* = 1.643
	225°	578.54	128.74	
Pair 7	45°	477.51	101.05	*t*(49) = 1.647, *p* = 0.106
	315°	472.21	107.65	
Pair 8	135°	568.69	126.59	*t*(49) = −1.433, *p* = 0.158
	225°	578.54	128.74	
Pair 9	135°	568.69	126.59	*t*(49) = 12.700, *p* < 0.001, *d* = 1.796
	315°	472.21	107.65	
Pair 10	225°	578.54	128.74	*t*(49) = 12.622, *p* < 0.001, *d* = 1.785
	315°	472.21	107.65	

Regarding accuracy rates, the repeated measures ANOVA revealed again a main effect of *Mirroring* (F(1, 49) = 15.438, *p* < 0.001, partial *η* ^2^ = 0.240) with lower hit rates in non‐mirrored (77.6% ± 14.3%) compared to mirrored trials (82.6% ± 11.3%). Further, a main effect of *Rotation* was observed (F(1.428, 69.956) = 155.897, *p* < 0.001, partial *η* ^2^ = 0.761), and, again, the interaction between factors *Mirroring* and *Rotation* was significant (F(2.092,102.525) = 23.445, *p* < 0.001, partial *η* ^2^ = 0.324). Again, these effects violated the assumption of sphericity and a Greenhouse–Geisser correction was applied (*ε* for Rotation = 0.357; *ε* Mirroring × Rotation = 0.523).

**FIGURE 3:**
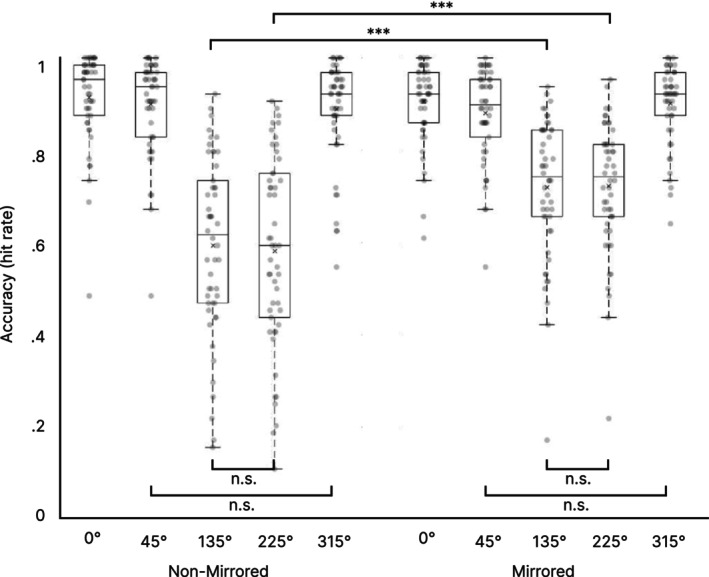
Behavioral accuracy results. Boxplots of hit rates from the Mental Rotation Task are shown for non‐mirrored and mirrored trials for all rotation angles. For reasons of clarity, only significant differences between conditions and non‐significant differences within conditions are shown. ***p < 0.001, n.s. = non‐significant. Centerline = median; × = mean; box limits = upper and lower quartiles, whiskers = minimum and maximum within 1.5× interquartile range, dots = individual data points.

Post hoc paired‐sample *t*‐tests were conducted to evaluate differences between hit rates in different rotation angles. Again, a Bonferroni correction was applied. In the non‐mirrored condition, no significant differences were found between rotation angles 0° and 45°, and between equivalent angles 45°/315° and 135°/225°. All other comparisons were significant (see Figure 3 and Table 2). In the mirrored condition, hit rates were not significantly different between 0° and 45° and 315°, respectively. Further, equivalent angles 45°/315° and 135°/225° did not differ significantly. All other comparisons were significant (see Figure 3 and Table 2).

**TABLE 2:** Descriptive accuracy measures and paired‐sample t‐tests for non‐mirrored and mirrored trials (*N* = 50).

Non‐mirrored		Mean	SD	*t*‐test
Pair 1	0°	0.92	0.10	*t*(49) = 2.784, *p* = 0.008
	45°	0.90	0.10	
Pair 2	0°	0.92	0.10	*t*(49) = 12.996, *p* < 0.001, *d* = 1.838
	135°	0.59	0.19	
Pair 3	0°	0.92	0.10	*t*(49) = 12.433, *p* < 0.001, *d* = 1.758
	225°	0.58	0.21	
Pair 4	0°	0.92	0.10	*t*(49) = 3.809, *p* < 0.001, *d* = 0.539
	315°	0.89	0.11	
Pair 5	45°	0.90	0.10	*t*(49) = 13.569, *p* < 0.001, *d* = 1.919
	135°	0.59	0.19	
Pair 6	45°	0.90	0.10	*t*(49) = 12.286, *p* < 0.001, *d* = 1.737
	225°	0.58	0.21	
Pair 7	45°	0.90	0.10	*t*(49) = 1.429, *p* = 0.159
	315°	0.89	0.11	
Pair 8	135°	0.59	0.19	*t*(49) = 0.748, *p* = 0.458
	225°	0.58	0.21	
Pair 9	135°	0.59	0.19	*t*(49) = −13.513, *p* < 0.001, *d* = 1.911
	315°	0.89	0.11	
Pair 10	225°	0.58	0.21	*t*(49) = −12.577, *p* < 0.001, *d* = 1.779
	315°	0.89	0.11	
Mirrored		Mean	SD	*t*‐test
Pair 1	0°	0.91	0.09	*t*(49) = 3.253, *p* = 0.002
	45°	0.88	0.10	
Pair 2	0°	0.91	0.09	*t*(49) = 8.246, *p* < 0.001, d = 1.166
	135°	0.72	0.15	
Pair 3	0°	0.91	0.09	*t*(49) = 8.334, *p* < 0.001, *d* = 1.179
	225°	0.72	0.14	
Pair 4	0°	0.91	0.09	*t*(49) = 0.585, *p* = 0.561
	315°	0.90	0.08	
Pair 5	45°	0.88	0.10	*t*(49) = 7.719, *p* < 0.001, *d* = 1.092
	135°	0.72	0.15	
Pair 6	45°	0.88	0.10	*t*(49) = 7.546, *p* < 0.001, *d* = 1.067
	225°	0.72	0.14	
Pair 7	45°	0.88	0.10	*t*(49) = −2.594, *p* = 0.012
	315°	0.90	0.08	
Pair 8	135°	0.72	0.15	*t*(49) = −0.137, *p* = 0.892
	225°	0.72	0.14	
Pair 9	135°	0.72	0.15	*t*(49) = −8.505, *p* < 0.001, *d* = 1.203
	315°	0.90	0.08	
Pair 10	225°	0.72	0.14	*t*(49) = −8.708, *p* < 0.001, *d* = 1.232
	315°	0.90	0.08	

The comparison of hit rates of the same rotation angles in the non‐mirrored and mirrored condition revealed a reversed pattern of significance compared to the investigation of RTs. More specifically, hit rates did not differ in low demanding rotation angles (i.e., 0°, 45°, and 315°). However, in high demanding rotation angles, lower hit rates were found in non‐mirrored than in mirrored trials (135°: *t*(49) = −4.916, *p* < 0.001, *d* = −0.965; 225°: *t*(49) = −4.810, *p* < 0.001, *d* = −0.680), which explains the interaction effect mentioned above.

### 3.2 Neurophysiological Results of Mental Rotation

The following analysis of EEG data focuses on the differences between the most extreme rotation angles (i.e., 0° and 135°/225°). To reduce complexity of the analyses, we pooled the data regarding the factor mirroring. Since behavioral results did not show significant differences between RTs and hit rates in trials with rotation angles 135° and 225°, we only analyzed data from rotation angle 135°.

#### 3.2.1 Oscillatory Signatures of Mental Rotation

**FIGURE 4:**
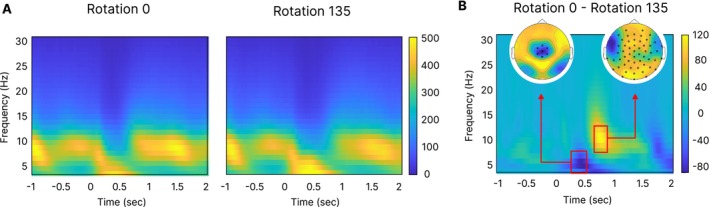
Time‐frequency results. Results of the (A) time‐frequency analysis for the two rotation angles of interest (i.e., 0° and 135°). Data were pooled regarding the factor mirroring. (B) shows the power differences between the two rotation conditions and including the results of the cluster‐based permutation tests (see topographies) in the according time windows and frequencies. Significant clusters are marked with small asterisks (*).

A time‐frequency analysis showed effects between rotation angle 0° and 135° in both the theta and alpha frequency regions (Figure 4A). An increase in theta power was observed from ~150 to 750 ms after stimulus onset. During the same time window, a decrease in alpha power was observed. The calculation of power difference (Figure 4B) showed a negative power difference (i.e., higher power in trials with rotation 135) for theta around ~250–500 ms, while a positive power difference was evident for alpha between ~600–800 ms. The cluster‐based permutation tests showed significant effects for both frequency bands in these time windows (see topographies). While theta power was greater in trials with a high demanding rotation angle (i.e., 135°), alpha power was lower in these trials compared to trials with low demanding rotation angle (i.e., 0°).

#### 3.2.2 Differences in Mid‐Frontal Theta and Fronto‐Temporal Alpha Power During High Demanding Mental Rotation

We used DICS beamforming to examine variations between conditions at the source level following the time‐frequency analysis and the evaluation of important changes at the sensor level (see the methods section for a further explanation). Since only a negative cluster in the theta frequency and a positive cluster in the alpha frequency band were significant on the sensor level, results from contrasts on the source level will only be interpreted following this pattern.

For the contrast of rotation angles (0° vs. 135°), we calculated the absolute difference between conditions by subtracting power values of condition 135° from 0° (i.e., x1−x2) to account for absolute power differences and subsequent used the DBSCAN method for clustering. Time windows for DICS input were based on visual inspection of the difference resulting in the time‐frequency analysis (see Figure 4B). To guarantee at least three complete cycles per frequency we chose different time windows for theta and alpha around the observed power differences. The calculation was as follows: 3 cycles in the lowest theta frequency (4 Hz) require 0.75 s (1 s/4 Hz × 3), while 3 cycles in the lowest alpha frequency (8 Hz) require 0.375 s (1 s/8 Hz × 3). The time window around the observed theta power difference was thus set to [0.2 0.95], and [0.6 1] was set for alpha.

**FIGURE 5:**
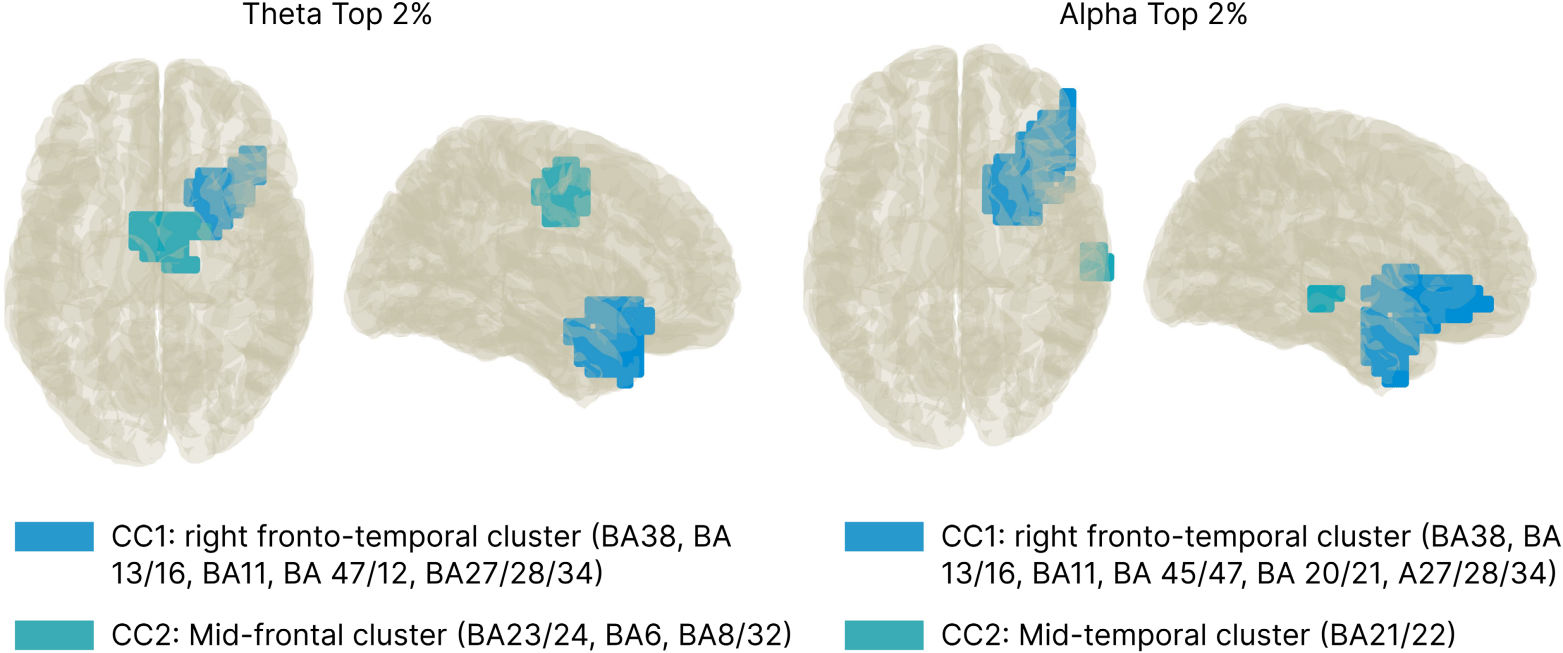
Contrast analysis of source activity. Absolute contrast activity between rotation angle 0° and 135° for theta and alpha band activity (top 2% active voxels). Colors indicate contrast clusters (CCs).

Figure 5 and Table 3 show the outcomes (i.e., brain regions from AAL and associated Brodmann areas [BAs]) of the top 2% of active voxels. For the theta frequency band, we found two CCs with higher theta power during low (i.e., 0°) compared to high demanding (i.e., 135°) mental rotation trials in inferior frontal (BA 11/12/47) and anterior temporal regions (BA 38 and BAs 20/21) together with the insula (BA 13/16), hippocampal (BA 27/28/34), and mid‐frontal (i.e., cingulate and supplemental motor) areas (BA 23/24 and BA 6). Within the alpha frequency band, 1 CC with stronger alpha power in low vs. high demanding condition was found, indicating lower alpha power in fronto‐temporal areas encompassing the inferior frontal gyrus (BA 11/45/47), the temporal pole (BA 38) and the insula (BA 13/16), hippocampal areas (BA 27/28/34), and parts of the basal ganglia during mental rotation (i.e., CC1). Another, smaller cluster (CC2) showed stronger alpha power in the middle temporal gyrus (BA 21/22) during high‐ compared to low‐demanding mental rotation efforts.

**TABLE 3:** Contrast clusters (CCs) for the contrasts between rotation conditions 0° and 135°.

AC		Contrast	Brain regions	Brodmann area (BA)
Theta				
1	Right fronto‐temporal	0 > 135	**Temporal pole**	BA 38
			**Insula**	BA 13/16
			**Inf frontal Gyrus**	BA 11; BA 47/12
			**Putamen**	—
			**Amygdala**	—
			Hippocampus	—
			**Parahippocampal Gyrus**	27/28/34
2	Mid‐frontal	0 > 135	**Middle Cingulate Cortex**	BA 23/24
			**Supplemental Motor Area**	BA 6
			Sup Frontal Gyrus	BA 8/32
Alpha				
1	Right fronto‐temporal	0 > 135	**Inf frontal Gyrus**	BA 11; BA 45/47
			**Temporal pole**	BA 38
			**Inf/Mid Temporal Gyrus**	BA 20; BA 21
			**Insula**	BA 13/16
			**Amygdala**	—
			Hippocampus	—
			**Parahippocampal Gyrus Putamen**	27/28/34
			**Pallidum**	—
2	Mid‐temporal	135 > 0	**Mid/**Sup **temporal Gyrus**	BA 21; BA 22

*Note:* Bold regions = more than 5 voxels. 0 = rotation angle 0°, 135 = rotation angle 135°.

#### 3.2.3 Network Architecture During Low and High Demanding Mental Rotation in Theta and Alpha

In preparation for the connectivity (i.e., nCREANN) analysis of underlying network architecture during different rotation conditions, we conducted three separate DICS beamforming analysis with subsequent voxel clustering (again top 2%) using the DBSCAN algorithm for both, theta and alpha frequency bands.

**FIGURE 6:**
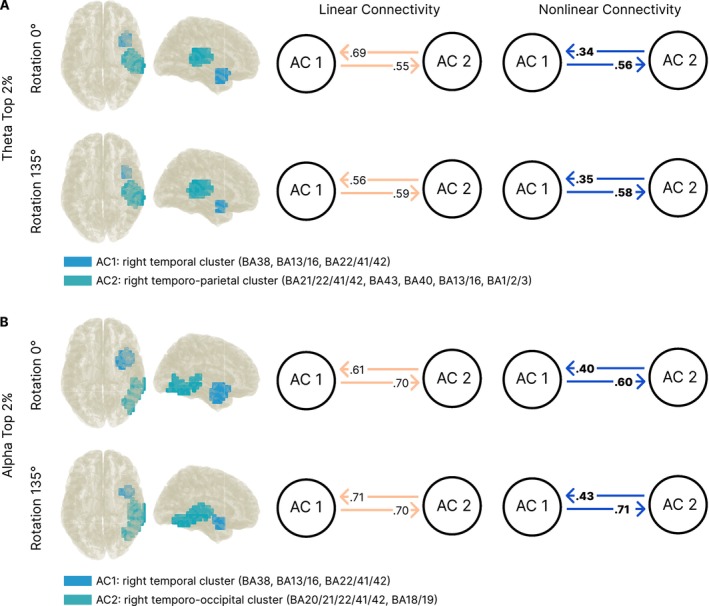
Directed connectivity in the (A) theta and (B) alpha frequency band. Results of the nCREANN algorithm for the average surrogate masked values across all subjects (i.e., on a group level) in both frequency bands. Activity clusters (ACs) for both rotation conditions are shown on the left side. Single non‐self connectivities for each condition from linear (orange) and nonlinear (blue) analyses are shown on the right side. Significant differences (corrected *p* < 0.05) between the directed connections from and to an AC are indicated by bold numbers.

Within each frequency band, regions of ACs largely overlapped (see Figure 6A,B). Table 4 show regions and according BAs for all active voxels in the separate rotation conditions. Voxels that were only found in one rotation condition are marked with (+) or (#). Further, we found similar right‐hemispheric activation patterns for both frequency bands. Especially AC1 showed overlapping activation in the theta and alpha network regarding anterior temporal regions (including the temporal pole, the superior temporal gyrus, and the insula). However, the extent of active voxels differed between frequency bands. Regarding AC2, again activation in (more posterior) temporal regions (BA 21/22/41/42) was observed in both frequency bands. However, the theta network additionally showed parietal activation (i.e., SMG and Heschl gyrus; BA 40 and BA 41) while the alpha network showed activation in the inferior occipital gyrus (BA 17/18).

**TABLE 4:** Activity clusters (ACs) for single rotation conditions 0° and 135°.

Theta ACs		Brain regions	Brodmann areas
**1**	**Right temporal**	**Temporal pole**	BA 38
		**Insula**	BA 13/16
		Superior temporal gyrus	BA 22/41/42
		Amygdala	—
		Middle temporal gyrus+	BA 21
		Inferior orbitofrontal gyrus+	BA 11
		Olfactory cortex+	BA 20/28/34
**2**	**Right temporo‐parietal**	**Mid/sup temporal gyrus**	BA 21, BA 22/41/42
		**Heschl gyrus**	BA 41
		**Subcentral gyrus**	BA 43
		**Supramarginal gyrus**	BA 40
		Insula	BA 13/16
		Postcentral gyrus	BA 1/2/3

Alpha ACs		Brain regions	Brodmann areas
1	Right temporal	Temporal pole	BA 38
		Superior temporal gyrus	BA 22/41/42
		Insula	BA 13/16
		Amygdala	—
		Hippocampus#	—
		Inf/mid temporal gyrus#	BA20, BA21
		Fusiform gyrus#	BA 20
		Parahippocampal gyrus#	BA 27/28/34
		Putamen#	—
		Olfactory cortex#	BA 20/28/34
		Inferior orbitofrontal gyrus#	BA 11
2	Right temporo‐occipital	Inf/mid/sup temporal gyrus	BA 20, BA21, BA 22/41/42
		Inferior occipital gyrus	BA 18/19
		Middle occipital gyrus+	BA 18
		Heschl gyrus#	BA 41
		Fusiform gyrus#	BA 20
		Subcentral gyrus#	BA 43

*Note:* + only in rotation angle 0°, # only in rotation angle 135°. Bold regions = more than 5 voxels. 0 = rotation angle 0°, 135 = rotation angle 135°.

The nCREANN analysis showed excellent performance measures (see Tables S1 and S2) and revealed directed connectivity values for the two ACs derived from the DICS/DBSCAN pipeline. Figure 6 shows the patterns of directed connectivity within the theta and alpha frequency bands for the rotation angles 0° and 135°. Within both networks, linear directed connectivity did not show asymmetries after FDR correction (*p* > 0.05) for both frequency bands. However, in the nonlinear model of directed connectivity, stronger directed information transfer was observed from AC1 to AC2 than vice versa. This was the case in the theta network for both rotation conditions (0°: *t*(49) = 3.61, *p* < 0.001, *p*_corr_ < 0.001, *d* = 0.51; 135°: *t*(49) = 2.90; *p* = 0.006, *p*_corr_ = 0.006, *d* = 0.41), as well as in the alpha network (0°: *t*(49) = 2.60, *p* = 0.012, *p*_corr_ = 0.012, *d* = 0.37; 135°: *t*(49) = 3.57, *p* < 0.001, *p*_corr_ = 0.002, *d* = 0.50). For details, see Tables S3 and S4.

**FIGURE 7:**
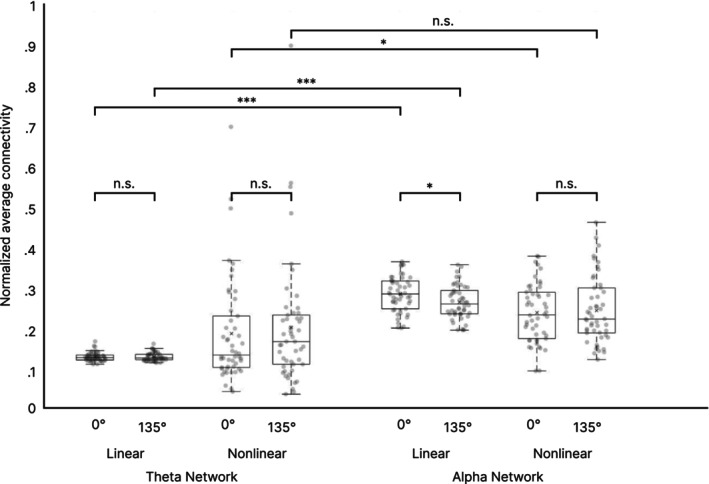
Averaged network connectivity in the theta and alpha frequency band. ***p < 0.001, *p < 0.05, n.s. = non‐significant. Centerline = median; × = mean; box limits = upper and lower quartiles, whiskers = minimum and maximum within 1.5× interquartile range, dots = individual data points.

Figure 7 shows linear and nonlinear average network connectivities for rotation angles 0° and 135° in both frequency bands. A significant difference in average network connectivity was only between rotation angles 0° and 135° in the linear alpha network (*t*(49) = −26.454, *p* = 0.011) with higher values during the low (0.29 ± 0.04) compared to high demanding (0.27 ± 0.04) mental rotation angle. No significant differences were found for nonlinear values in the alpha network (*p* = 0.661), nor in the theta network linear: *p* = 0.999; nonlinear (*p* = 0.602).

Comparisons of normalized averaged connectivity between conditions in the two frequency bands showed significant differences between linear averaged connectivity between theta and alpha for the low demanding rotation condition (*t*(49) = −25.292, *p* < 0.001) and the high demanding condition (*t*(49) = −23.367, *p* < 0.001), with higher averaged connectivity values in the alpha network (low: 0.29 ± 0.04; high: 0.27 ± 0.04) than in the theta network (low: 0.13 ± 0.01; high: 0.13 ± 0.01). Further, a significant difference was found between nonlinear averaged connectivity in the low demanding condition between alpha and theta (*t*(49) = 2.147, *p* = 0.037) with again higher values in the alpha (0.24 ± 0.07) compared to the theta network (0.19 ± 0.13). Nonlinear average connectivity did not differ in the high demanding condition between alpha and theta (*p* = 0.052).

## 4 Discussion

Understanding how the brain transforms internal representations is central to cognitive control. The current study aimed at investigating the role of underlying directed connectivity profiles in theta and alpha band activity between cortical regions during mental rotation processes, which rely on maintenance and retrieval of working memory content. Here, we show that mental rotation is associated with nonlinear, directed communication between anterior conceptual–control and posterior perceptual–motor systems. These results suggest that oscillatory networks in the theta and alpha bands may provide a dynamic bridge between control, memory, and perception, consistent with a role in the transformation of abstract goals into internal spatial operations.

The data replicated previous behavioral results, showing a gradual increase of RTs and errors with increase of rotation angles (Butler et al. 2006; Gogos et al. 2010; Varriale et al. 2018; Yu et al. 2023; Zhao et al. 2022), indicating the robustness of mental rotation effects of the paradigm. In particular, RTs reached a “turning point” at a rotation degree of 135° and 225°, respectively, whereby RTs did not differ between these rotation angles in non‐mirrored trials, nor in mirrored trials. Further, RTs were again faster after this “turning point”, showing similar RTs for rotation angles 315° and 45° (see also (Kung and Hamm 2010; Searle and Hamm 2017)), which is in line with the idea that mental rotation occurs through the shortest angle (Kung and Hamm 2010). The same pattern was observed for error rates (i.e., increasing till “turning point” and subsequently decreasing). Interestingly, significant higher error rates were observed for the most demanding rotation angles (i.e., 135° and 225°) in non‐mirrored trials compared to mirrored trials, which was also shown before (Kung and Hamm 2010; Núñez‐Peña and Aznar‐Casanova 2009; Paschke et al. 2012).

### 4.1 Functional Neuroanatomical and Neurophysiological Findings

Time‐frequency analyses showed higher theta power over central electrodes together with a broad lower alpha power during high‐demand mental rotation (135°) relative to 0° (Figure 4). This is in line with previous research on cognitive control processes regarding oscillatory power, since an increase in theta power is suggested to reflect demands of cognitive control (Cavanagh et al. 2012; Cavanagh and Shackman 2015; Hsieh and Ranganath 2014; Jensen and Tesche 2002; Lockhart et al. 2019; Widge et al. 2019). Further, a decrease in alpha power was shown before during tasks of increasing working memory demands and mental rotation (Nikolaev and Anokhin 1998; Pineda 2005).

Subsequent source localization of the contrast between conditions (0° vs. 135°) revealed higher theta power during low demanding mental rotation in two CCs formed of the top 2% of the difference between oscillatory power in both conditions (Figure 5). These CCs were: (i) an anterior temporal–insular–inferior frontal cluster, which is associated with conceptual integration and estimation of control demands (Jiang et al. 2015; Liakakis et al. 2011; Mayer et al. 2025; Rice et al. 2015; Schaum et al. 2021; Swick et al. 2011; Wager et al. 2005), and (ii) a mid‐frontal cingulate/supplemental motor area (SMA) cluster that aligns with canonical theta generators involved in motor simulation and internal action planning (Cavanagh and Frank 2014; Cohen 2014; Fukumoto et al. 2021; Hardwick et al. 2018; Nigbur et al. 2011; Zacks 2008). In the context of prior work linking theta to control and working memory updating (Cavanagh et al. 2012; Cavanagh and Frank 2014; Cavanagh and Shackman 2015; Hsieh and Ranganath 2014; Jensen and Tesche 2002; Lockhart et al. 2019; Widge et al. 2019), the theta reduction in the high demanding rotation angle could reflect a reconfiguration away from sustained frontal control once visuospatial transformation processes dominate. In other words, the theta decrease in these CCs with higher rotation demand might mirror a shift of processing emphasis toward posterior visuospatial implementation, once the action concept is established. In the alpha band, higher power was localized within fronto‐temporal/ventral‐stream regions, namely the inferior frontal gyrus (IFG; BA 11/45/47), anterior temporal pole (BA 38), insula (BA 13/16), and hippocampal structures (BA 27/28/34) during low demanding mental rotation. These structures are known to support object recognition and mnemonic maintenance (Kravitz et al. 2013; Zimmer 2008). Here, the alpha decrease during high demanding mental rotation (see also Chen et al. 2013; Gardony et al. 2017) might reflect the engagement of larger neural populations (Pineda 2005), as distributed activity facilitates dynamic and efficient coding of information (Hanslmayr et al. 2012, 2016) as well as memory encoding and retrieval (Hanslmayr et al. 2012, 2014; Klimesch et al. 1997). On the other hand, higher alpha power during high demands of mental rotation in a second, smaller mid‐temporal cluster (CC2) might reflect the active shielding of the to‐be‐rotated object representation, since an increase in alpha power has been suggested to facilitate top‐down inhibitory gating and shielding of task‐relevant representations (Elmers et al. 2024; Foxe and Snyder 2011; Jamous et al. 2024; Klimesch 2012; Konjusha et al. 2023), thus stabilizing the internal object template during rotation.

Taken together, visuospatial transformations in working memory, as examined through mental rotation, may involve a shift in the relative contribution of theta‐ and alpha‐band processes. As rotation demands rise, frontal theta‐related activity appears reduced, while alpha‐band modulations may reflect both enhanced encoding‐related recruitment (i.e., alpha decrease) and the stabilization or shielding of the rotated representation (i.e., alpha increase). The directed connectivity analyses are consistent with an anterior‐to‐posterior organization of these inferred functional interactions, but they do not by themselves establish a causal “hand‐off” between frequency bands.

### 4.2 Directed Connectivity in Theta and Alpha Bands

The nCREANN analysis revealed distinct directed connectivity profiles in the theta and alpha frequency bands, suggesting differentiated patterns of cortical communication during low and high demands of mental rotation. Across both frequency networks, two right‐lateralized activity clusters (ACs) emerged from the DICS/DBSCAN source pipeline: AC1 showed a great overlap between frequency bands, encompassing the anterior temporal lobe (ATL), insula, IFG, and superior temporal gyrus (STG). AC2, on the other hand, included posterior temporo‐parietal regions in the theta network, and temporo‐occipital regions in the alpha network (see Figure 6). In particular, AC2 in the theta network encompassed voxels located in the temporo‐parietal junction (TPJ), which has been repeatedly been associated with WM monitoring and updating (Alagapan et al. 2019; du Boisgueheneuc et al. 2006; Christophel et al. 2017; Dippel et al. 2016; Eich et al. 2023; Geng and Vossel 2013; Nissim et al. 2016; Rypma et al. 1999; Tarantino et al. 2017; Vallesi et al. 2022; Wang, He, et al. 2019), including task‐set information (Chmielewski et al. 2017; Dosenbach et al. 2008), as well as response selection (Eich et al. 2023; Vallesi et al. 2022). In the alpha network on the other hand, regions of the ventral visual pathway including the temporal and inferior occipital gyrus formed AC2. These regions are important for complex visual perception, object recognition and imagination (Zimmer 2008), stimulus–response integration (Dibbets et al. 2010; Goghari and MacDonald 2009), and WM maintenance (Bluhm et al. 2011; Park et al. 2011; Xie et al. 2019). Taken together, ACs found in the single conditions are associated with different processes of mental rotation, including visual recognition and encoding of an object, the manipulation and maintenance of its rotated representation in the working memory, and subsequent decision‐making processes (i.e., deciding whether an object is mirrored or not). The right‐lateralization effect is in line with previous EEG research reporting higher activation and earlier onset of right compared to left parietal negativity during mental rotation (Milivojevic et al. 2009). Further, MRI and lesion data confirmed the importance of right parietal regions for mental rotation processes (Gogos et al. 2010; Tomasino et al. 2003). Since the stimuli used in the current study were rather simple, the right lateralized activation might also reflect a rather holistic than complex and analytic processing style (Jansen‐Osmann and Heil 2007).

While linear models showed no directional asymmetries, nonlinear directed connectivity analyses indicated a reliable anterior‐to‐posterior asymmetry in directed information transfer from AC1 to AC2 in both the theta and alpha bands across rotation conditions (theta: all *p* < 0.01; alpha: all *p* < 0.01; see Figure 6 and Tables S1 and S2). In the theta band, this anterior‐to‐posterior pattern originating in the ATL, IFG, and insula and targeting the TPJ is consistent with top‐down coordination and monitoring during the updating of visuospatial working memory representations (Alagapan et al. 2019; du Boisgueheneuc et al. 2006; Christophel et al. 2017; Dippel et al. 2016; Eich et al. 2023; Geng and Vossel 2013; Nissim et al. 2016; Rypma et al. 1999; Tarantino et al. 2017; Vallesi et al. 2022; Wang, He, et al. 2019). The combination of reduced theta power and evident directional coupling suggests that cognitive control may become less globally synchronized but more selectively routed during high compared to low demands, consistent with more focused transfer of control‐related signals to posterior parietal sites involved in transformation and comparison of object orientations. Functionally, theta‐band connectivity may provide a scaffold through which anterior conceptual control regions bias posterior spatial‐motor systems, thereby helping maintain representational coherence during demanding transformations (Buzsáki 2006; Cavanagh and Frank 2014). In contrast, the alpha‐band network exhibited stronger overall connectivity values and a two‐fold modulation in power during mental rotation, coupled with directed information flow from fronto‐temporal to occipito‐temporal regions. This pattern aligns with the role of alpha band activity in gating and stabilizing perceptual representations (Elmers et al. 2024; Foxe and Snyder 2011). The directed alpha connectivity from the IFG, temporal pole, and insula toward posterior visual areas may reflect the transmission of mnemonic templates and perceptual predictions. This anteriorly driven alpha influence may complement theta‐related control dynamics, together suggesting a dynamic cross‐frequency network architecture during spatial reasoning.

Taken together, the observed ATL‐centered connectivity pattern is consistent with a role of anterior temporal–frontal–insular regions in coordinating processes relevant for stimulus–response binding, maintenance of task‐specific information, and working‐memory monitoring/updating during mental rotation.

### 4.3 Limitations

The current study used a data‐driven beamforming approach to define regions of interest (ROIs; i.e., DICS beamforming results), which may have resulted in a lack of spatial accuracy. Further, the threshold used for voxel selection in DBSCAN clustering is another methodological factor that must be taken into account. We selected the top 2% of active voxels, as this threshold offered a reasonable compromise between interpretability and spatial specificity (i.e., between small, scattered clusters and large, diffuse clusters with low spatial resolution). However, the choice of thresholds also influences the cluster configurations, which could make replication approaches more difficult. Future studies could therefore use theory‐driven definitions of ROIs as input for the nCREANN algorithm. However, this could also come with caveats, as most research on ROIs in cognition comes from MRI research, which is methodologically different. Another methodological objection is that the neurophysiological analyses were conducted on pooled data, whereby mirroring effects were not taken into account. While RTs did not differ between mirrored and non‐mirrored trials in the high demanding condition, accuracy was significantly higher in mirrored rather than non‐mirrored trials in high demanding mental rotation. For the low demanding condition, this pattern was reversed. Additional time‐frequency analyses further showed small, but significant effects between mirrored and non‐mirrored trials (see Figure S3). Future studies should keep this in mind in order to address the potential effects of mirroring.

### 4.4 Conclusions

In summary, the study suggests that the transformation of internal representations in working memory is associated with directional communication across a distributed cortical hierarchy. Directed connectivity analyses indicated that anterior temporal–frontal–insular regions show stronger anterior‐to‐posterior interactions with posterior visuospatial cortices, consistent with a role in integrating conceptual, mnemonic, and perceptual codes during mental rotation. These findings further suggest frequency‐specific modes of communication, with theta‐band interactions being consistent with control and updating processes, and alpha‐band interactions being consistent with representational stabilization.

## Author Contributions

All authors had full access to the data, gave final approval for publication, and agreed to be held accountable for the work performed therein. JE: Conceptualization, Investigation, Methodology, formal analysis, Visualization, Writing – original draft; MM: Formal Analysis, Methodology, Software, Visualization, Writing – original draft; CB: Conceptualization, Funding acquisition, Methodology, Resources, Supervision, Writing – original draft. All authors approved the final version of the manuscript.

## Funding

This work was supported by a Grant from the Deutsche Forschungsgemeinschaft SFB 940 to C.B.

## Ethics Statement

All participants provided written informed consent and received a financial reimbursement for their participation in the study. The ethics committee of TU Dresden approved this study.

## Consent

The authors have nothing to report.

## Conflicts of Interest

The authors declare no conflicts of interest.

## Supporting information


**Figure S1:** Flowchart of analyzing pipeline. Top half: Preparatory steps before applying Automagic are shown in grey, Automagic steps are shown in light blue, while post‐processing steps are shown in light green. Lower half: Representation of the time‐frequency, beamforming, and connectivity analyses. After time‐frequency analyses of theta [0.2–0.95] and alpha [0.6–1.0] power, cluster‐based permutation tests were conducted. Two lines of analyses followed: contrast analyses with specified time‐windows of interest, and connectivity analyses with nCREANN in a target‐locked time window [0–1.0].
**Figure S2:** Plots of AIC(p) and SBC(p) curves as functions of model order p. The “elbow” point in the curve (marked with red circle) was taken as the optimal model order. (A) shows AIC(p) and SBC(p) plots for theta frequency band and (B) for alpha frequency band.
**Figure S3:** Time‐frequency results for non‐mirror and mirror trials on the left side and differences on the right side. (A) shows results for the 0° rotation condition and (B) for the and 135° rotation condition. Cluster‐based permutation testing showed significant results for theta and alpha during 0° rotation, while only alpha effects were found during the 135° rotation.
**Table S1:** Performance Measures of nCREANN for theta.
**Table S2:** Performance Measures of nCREANN for alpha.
**Table S3:** Connectivity values of theta network.
**Table S4:** Connectivity values of alpha network.
**Table S5:** Comparison of normalized connectivities between 0° and 135° of theta network.
**Table S6:** Comparison of normalized connectivities between 0° and 135° of alpha network.

## Data Availability

Raw data can be found in the Open Access Repository and Archive (OPARA) from TU Dresden after acceptance. The numerical source data for Figures 2 and 3 can be found in logfiles and tables under the name “01_Behavioral data.zip”, numerical source data for Figures 4, 5, 6, 7 can be found under the name “02_Preprocessed EEG data.zip”.
