# Correction to “Directed Communication in Theta and Alpha Networks Supports Content Handling in Working Memory”

**DOI:** 10.1002/hbm.70613

**Published:** 2026-07-14

Elmers, J. , M. Mückschel , and C. Beste . 2026. “Directed Communication in Theta and Alpha Networks Supports Content Handling in Working Memory.” Human Brain Mapping 47, no. 7: e70537.42057539 10.1002/hbm.70537PMC13129412

The affiliation of the authors was incomplete and has been corrected to include the university name. The correct affiliation is: “Department of Child and Adolescent Psychiatry, Faculty of Medicine, Cognitive Neurophysiology, TUD Dresden University of Technology, Dresden, Germany.”

The online version of this article has been corrected accordingly.

We apologize for this error.

