# Supplementary material for: Directed Communication in Theta and Alpha Networks Supports Content Handling in Working Memory
Source: Hum Brain Mapp. 2026 Apr 29;47(7):e70537. doi: 10.1002/hbm.70537 (PMC13129412; doi:10.1002/hbm.70537)
Supplement: Supplementary file 1 — Figure S1: Flowchart of analyzing pipeline. Top half: Preparatory steps before applying Automagic are shown in grey, Automagic steps are shown in light blue, while post‐processing steps are shown in light green. Lower half: Representation of the time‐frequency, beamforming, and connectivity analyses. After time‐frequency analyses of theta [0.2–0.95] and alpha [0.6–1.0] power, cluster‐based permutation tests were conducted. Two lines of analyses followed: contrast analyses with specified time‐windows of interest, and connectivity analyses with nCREANN in a target‐locked time window [0–1.0]. Figure S2: Plots of AIC(p) and SBC(p) curves as functions of model order p. The “elbow” point in the curve (marked with red circle) was taken as the optimal model order. (A) shows AIC(p) and SBC(p) plots for theta frequency band and (B) for alpha frequency band. Figure S3: Time‐frequency results for non‐mirror and mirror trials on the left side and differences on the right side. (A) shows results for the 0° rotation condition and (B) for the and 135° rotation condition. Cluster‐based permutation testing showed significant results for theta and alpha during 0° rotation, while only alpha effects were found during the 135° rotation. Table S1: Performance Measures of nCREANN for theta. Table S2: Performance Measures of nCREANN for alpha. Table S3: Connectivity values of theta network. Table S4: Connectivity values of alpha network. Table S5: Comparison of normalized connectivities between 0° and 135° of theta network. Table S6: Comparison of normalized connectivities between 0° and 135° of alpha network. [file HBM-47-e70537-s001.docx]

# Supplements

## Analyzing pipeline


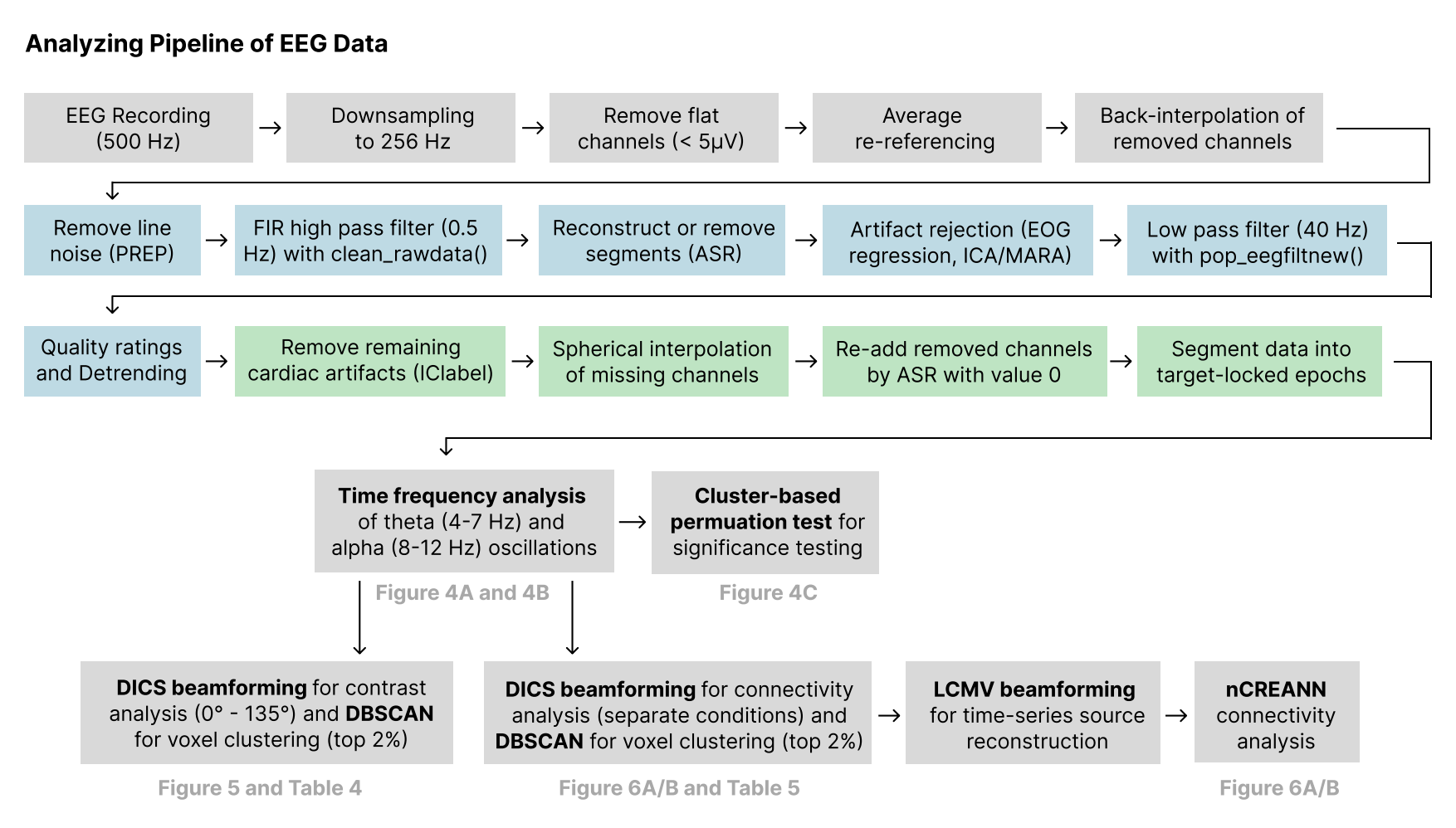


***Figure S1.*** *Flowchart of analyzing pipeline. Top half: Preparatory steps before applying Automagic are shown in grey, Automagic steps are shown in light blue, while post-processing steps are shown in light green. Lower half: Representation of the time-frequency, beamforming, and connectivity analyses. After time-frequency analyses of theta [0.2–0.95] and alpha [0.6–1.0] power, cluster-based permutation tests were conducted. Two lines of analyses followed: contrast analyses with specified time-windows of interest, and connectivity analyses with nCREANN in a target-locked time window [0–1.0].*

## Model Order for nCREANN input

*
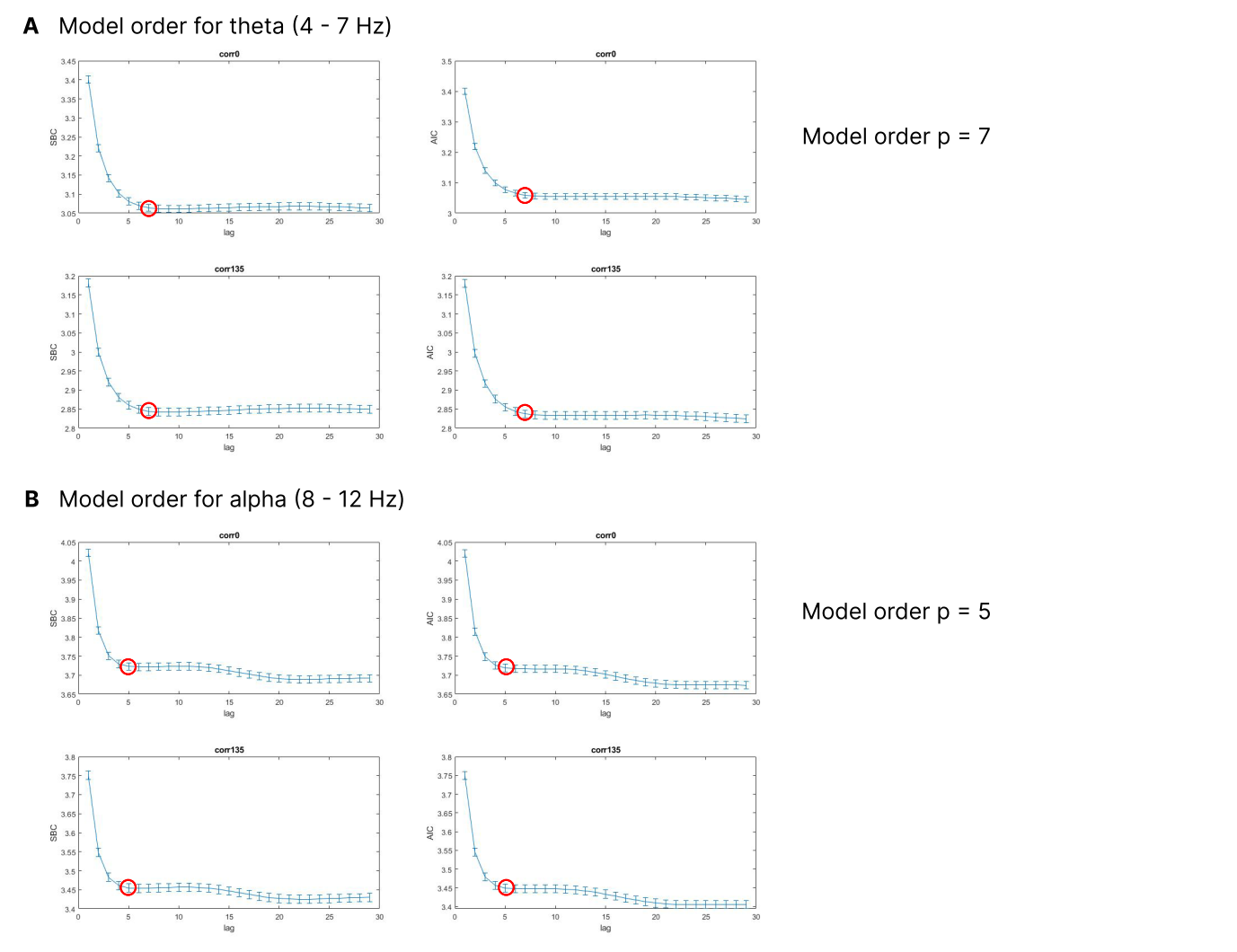
*

***Figure S2.*** *Plots of AIC(p) and SBC(p) curves as functions of model order p. The "elbow" point in the curve (marked with red circle) was taken as the optimal model order. A) shows AIC(p) and SBC(p) plots for theta frequency band and B) for alpha frequency band.*


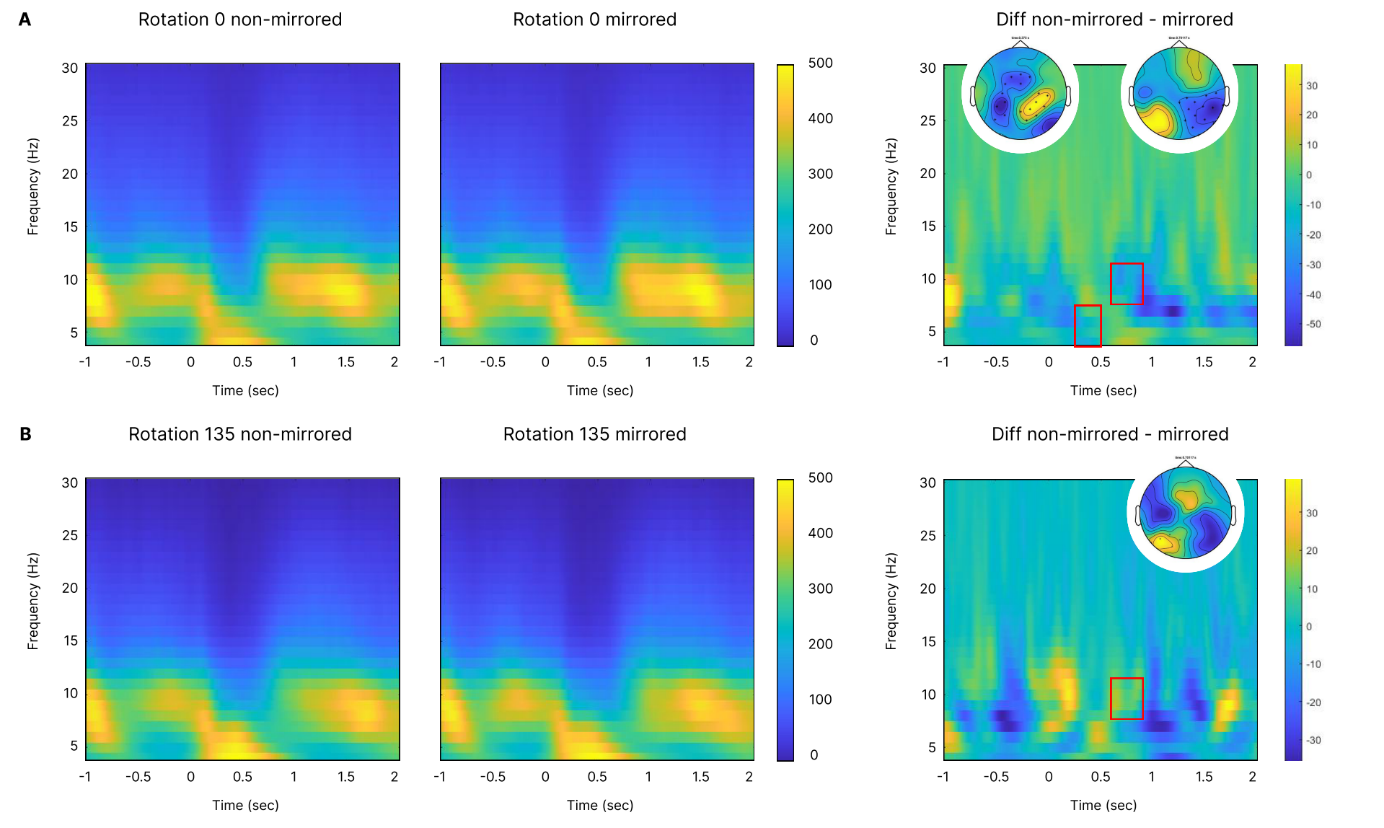


**Figure S3.** Time-frequency results for non-mirror and mirror trials on the left side and differences on the right side. A) shows results for the 0° rotation condition and B) for the and 135° rotation condition. Cluster-based permutation testing showed significant results for theta and alpha during 0° rotation, while only alpha effects were found during the 135° rotation.

## Quality Measures of nCREANN Analysis

We calculated performance measures of the nCREANN algorithm. The tables below show performance measures for training and test trials, and mean square errors (MSE) for both conditions. The analysis showed excellent quality measures for both theta and alpha networks (all > 98%; see tables below). Significant differences were observed in MSE. Nevertheless, the absolute MSE values were sufficiently small for both conditions (ranging from 3.5 to 5.1 %).

**Table S1.** Performance Measures of nCREANN for theta.

| **Quality Measure** | **Rotation 0 (Mean)** | **Rotation 0 (SD)** | **Rotation 135 (Mean)** | **Rotation 135 (SD)** | **0 vs 135**  **(p)** |
| --- | --- | --- | --- | --- | --- |
| **R2_train_AC_1** | 0.985 | 0.005 | 0.984 | 0.007 | 0.701 |
| **R2_train_ AC_2** | 0.984 | 0.006 | 0.983 | 0.008 | 0.676 |
| **err_MSE_train** | 0.039 |  | 0.045 |  | **0.021** |
| **R2_test_ AC_1** | 0.987 | 0.004 | 0.985 | 0.006 | 0.064 |
| **R2_test_ AC_2** | 0.984 | 0.007 | 0.985 | 0.007 | 0.506 |
| **err_MSE_test** | 0.043 |  | 0.051 |  | **0.007** |

**Table S2.** Performance Measures of nCREANN for alpha.

| **Quality Measure** | **Rotation 0 (Mean)** | **Rotation 0 (SD)** | **Rotation 135 (Mean)** | **Rotation 135 (SD)** | **0 vs 135**  **(p)** |
| --- | --- | --- | --- | --- | --- |
| **R2_train_AC_1** | 0.986 | 0.003 | 0.985 | 0.003 | 0.420 |
| **R2_train_ AC_2** | 0.985 | 0.003 | 0.984 | 0.003 | 0.432 |
| **err_MSE_train** | 0.035 |  | 0.038 |  | **0.015** |
| **R2_test_ AC_1** | 0.986 | 0.003 | 0.985 | 0.003 | 0.337 |
| **R2_test_ AC_2** | 0.985 | 0.003 | 0.984 | 0.003 | 0.468 |
| **err_MSE_test** | 0.042 |  | 0.045 |  | 0.080 |

## nCREANN connectivity statistics

**Table S3.** Connectivity values of theta network

| **Model** | **Condition** | **AC1** | **AC2** | **p** | **p_corr_** | **statistics** |
| --- | --- | --- | --- | --- | --- | --- |
| Normalized connectivity values | | | | | | |
| Linear | 0° | 0.55 ± 0.05 | 0.69 ± 0.05 | **0.045** | 0.091 | t(49) = -2.05; p = 0.045; d = 0.29 |
| Linear | 135° | 0.59 ± 0.05 | 0.56 ± 0.06 | 0.652 | 0.652 | t(49) = 0.45; p = 0.652; d = 0.06 |
| Nonlinear | 0° | 0.56 ± 0.06 | 0.34 ± 0.05 | **0.001** | **0.001** | t(49) = 3.61; p < .001; d = 0.51 |
| Nonlinear | 135° | 0.58 ± 0.06 | 0.35 ± 0.05 | **0.006** | **0.006** | t(49) = 2.90; p = 0.006; d = 0.41 |
| Raw non-normalized connectivity values | | | | | | |
| Linear | 0° | 0.02 ± 0.00 | 0.03 ± 0.00 | **0.018** | **0.037** | t(49) = -2.44; p = 0.018; d = 0.34 |
| Linear | 135° | 0.03 ± 0.00 | 0.02 ± 0.00 | 0.492 | 0.492 | t(49) = 0.69; p = 0.492; d = 0.10 |
| Nonlinear | 0° | 0.13 ± 0.02 | 0.06 ± 0.01 | **< 0.001** | **0.001** | t(49) = 3.75; p < .001; d = 0.53 |
| Nonlinear | 135° | 0.14 ± 0.02 | 0.07 ± 0.01 | **0.006** | **0.006** | t(49) = 2.85; p = 0.006; d = 0.40 |

**Table S4.** Connectivity values of alpha network

| **Model** | **Condition** | **AC1** | **AC2** | **p** | **p_corr_** | **statistics** |
| --- | --- | --- | --- | --- | --- | --- |
| Normalized connectivity values | | | | | | |
| Linear | 0° | 0.70 ± 0.04 | 0.61 ± 0.04 | **0.044** | 0.089 | t(49) = 2.06; p = 0.044; d = 0.29 |
| Linear | 135° | 0.70 ± 0.04 | 0.71 ± 0.04 | 0.819 | 0.819 | t(49) = -0.23; p = 0.819; d = 0.03 |
| Nonlinear | 0° | 0.60 ± 0.05 | 0.40 ± 0.05 | **0.012** | **0.012** | t(49) = 2.60; p = 0.012; d = 0.37 |
| Nonlinear | 135° | 0.71 ± 0.05 | 0.43 ± 0.05 | **0.001** | **0.002** | t(49) = 3.57; p < .001; d = 0.50 |
| Raw non-normalized connectivity values | | | | | | |
| Linear | 0° | 0.09 ± 0.01 | 0.08 ± 0.01 | **0.034** | 0.068 | t(49) = 2.18; p = 0.034; d = 0.31 |
| Linear | 135° | 0.08 ± 0.01 | 0.08 ± 0.01 | 0.925 | 0.925 | t(49) = -0.09; p = 0.925; d = 0.01 |
| Nonlinear | 0° | 0.12 ± 0.01 | 0.09 ± 0.01 | 0.093 | 0.093 | t(49) = 1.71; p = 0.093; d = 0.24 |
| Nonlinear | 135° | 0.16 ± 0.02 | 0.09 ± 0.01 | **0.004** | **0.008** | t(49) = 3.03; p = 0.004; d = 0.43 |

**Table S5.** Comparison of normalized connectivities between 0° to 135° of theta network

| **Model** | **ROIs** | **p** | **p_corr_** | **t** | **d** |
| --- | --- | --- | --- | --- | --- |
| Linear | AC1 > AC2 | 0.569 | 0.569 | -0.573 | 0.081 |
| Linear | AC2 > AC1 | 0.101 | 0.202 | 1.671 | 0.236 |
| Nonlinear | AC1 > AC2 | 0.870 | 0.897 | -0.164 | 0.023 |
| Nonlinear | AC2 > AC1 | 0.897 | 0.897 | -0.130 | 0.018 |

Note that the ROIs are largely overlapping, but their constituent voxels are not identical between 0° and 135°.

**Table S6.** Comparison of normalized connectivities between 0° to 135° of alpha network

| **Model** | **ROIs** | **p** | **p_corr_** | **t** | **d** |
| --- | --- | --- | --- | --- | --- |
| Linear | AC1 > AC2 | 0.969 | 0.969 | 0.039 | 0.005 |
| Linear | AC2 > AC1 | 0.076 | 0.152 | -1.812 | 0.256 |
| Nonlinear | AC1 > AC2 | 0.116 | 0.231 | -1.602 | 0.227 |
| Nonlinear | AC2 > AC1 | 0.617 | 0.617 | -0.503 | 0.071 |

Note that the ROIs are largely overlapping, but their constituent voxels are not identical between 0° and 135°.
